# An information-based approach to handle various types of uncertainty in fuzzy bodies of evidence

**DOI:** 10.1371/journal.pone.0227495

**Published:** 2020-01-13

**Authors:** Atiye Sarabi-Jamab, Babak N. Araabi

**Affiliations:** 1 School of Cognitive Sciences, Institute for Research in Fundamental Sciences (IPM), Tehran, Iran; 2 Control and Intelligent Processing Center of Excellence, School of Electrical and Computer Engineering, University of Tehran, Tehran, Iran; University of Padova, ITALY

## Abstract

Fuzzy evidence theory, or fuzzy Dempster-Shafer Theory captures all three types of uncertainty, i.e. fuzziness, non-specificity, and conflict, which are usually contained in a piece of information within one framework. Therefore, it is known as one of the most promising approaches for practical applications. Quantifying the difference between two fuzzy bodies of evidence becomes important when this framework is used in applications. This work is motivated by the fact that while dissimilarity measures have been surveyed in the fields of evidence theory and fuzzy set theory, no comprehensive survey is yet available for fuzzy evidence theory. We proposed a modification to a set of the most discriminative dissimilarity measures (smDDM)-as the minimum set of dissimilarity with the maximal power of discrimination in evidence theory- to handle all types of uncertainty in fuzzy evidence theory. The generalized smDDM (FsmDDM) together with the one previously introduced as fuzzy measures make up a set of measures that is comprehensive enough to collectively address all aspects of information conveyed by the fuzzy bodies of evidence. Experimental results are presented to validate the method and to show the efficiency of the proposed method.

## 1. Introduction

Dempster-Shafer theory (DST) or evidence theory is accepted as a flexible framework to model various processes of quantitative reasoning and decision making under uncertainty [1] [2] [3]. It is widely used in practical applications such as belief function approximation [4] [5], regression analysis [6] [7], sensor reliability evaluation [8] [9], risk analysis [10], sensor fusion [11], pattern classification [12] [13], and evidential clustering [14], where DST framework could handle different types of non-specificity, and conflict during modeling under uncertainty.

In order to effectively manage DST to handle fuzzy information in evidential reasoning, several generalization methods of DST to fuzzy sets have been proposed [15] [16] [17] [18] [19] [20]. Computing a Fuzzy Body of Evidence (FBoE) can quantify different sort of uncertainties, such as imprecision, discord and degree of confidence [21].

In all applications, measuring the difference is embedded in the body of the respective algorithms in one way or another. By choosing a suitable set of dissimilarity measures, the overall performance of the algorithm is improved. In our previous study, [22], we proposed a set measures to handle non-specificity and conflict, and we employed it for two applications. However, the measuring of fuzziness was not included, and it could not use for fuzzy framework. Thus, measuring the difference between the two FBoEs is a challenging task as the dissimilarity measures have a central role in the body of the algorithms.

In fuzzy set theory, Bloch proposed a detailed survey of distances between fuzzy sets, where they were used in image processing applications [23]. Also, de Campos *et al*. proposed a method to find distances between fuzzy measures based on associated probability distributions [24]. In DST, many works on measuring the distance between belief functions have emerged. In our previous study [22], we proposed a framework for comprehensive assessment of dissimilarity between two BoEs. The outcome was the set of most discriminative dissimilarity measures (smDDM) representing the minimal set of dissimilarity measures needed for an overall evaluation of the differences between two BoEs. Although dissimilarity measures in the field of both evidence theory and fuzzy set theory have been studied separately, no comprehensive survey is yet available for fuzzy evidence theory to handle all types of uncertainties.

Motivated by this fact, we investigate a method to comprehensively address all types of uncertainty. By modifying the set of discriminative dissimilarity measures (smDDM), we follow an information-based approach to propose such a method. To validate the proposed approach, it is tested through experimentation which shows a good agreement with experimental data.

## 2. Background

Dempster-Shafer Theory (DST) has been applied to quantitative reasoning and decision-making cases under uncertainty. Whereas the fuzzy DST as an extension to DST, is used to manage imprecise and vague information in evidential reasoning. In the following we review the general concepts of the two aforementioned theories and their corresponding basis.

### 2.1 Dempster-Shafer Theory (DST)

DST, or evidence theory, assigns mass values to the subsets of Frame of Discernment (FoD), instead of its elements. Let Θ be the FoD as a finite discrete set with N hypotheses, Θ = {ω_1_, …, ω_*N*_}, a mass function *m*(.), so called a Basic Probability Assignment (BPA), is defined as:

$$P_{\Theta }\to [0,1],\;\sum_{A\in P_{\Theta }}m(A)=1,\;m(A)\geq 0,\;\forall A\in P_{\Theta }$$ (1)

where *P*_Θ_ is the power set of Θ. All subsets of Θ with non-zero mass values are called Focal Elements (FEs), and all non-zero mass values (i.e. FEs along with their mass values) form a BoE. A BoE with *n* FEs is defined as:

$$\left\{A_{1},A_{2},\cdots ,A_{n}\},\{m_{1},m_{2},\cdots ,m_{n}\right\}$$

$$\phi \neq A_{j}\subset \Theta ,m_{j}>0,\sum m_{j}=1$$ (2)

Given the mass functions of a BPA, a belief function Bel, and a plausibility function, Pl are introduced as [1] [2]:

$$Bel:P_{\Theta }\to [0,1],Bel(B)=\sum_{A\subseteq B,A\neq \phi }m(A),\forall B\subseteq \Theta$$ (3)

$$Pl:P_{\Theta }\to [0,1],Pl(B)=\sum_{A\subseteq \Theta ,A\cap B\neq \phi }m(A),\forall B\subseteq \Theta$$ (4)

To aggregate several pieces of evidence, the combination rule plays a crucial role. Combination of the two independent bodies of evidence with their corresponding mass functions *m*_1_(.) and *m*_2_(.) is given by the Dempster’s rule of combination as defined in [1].

$$m_{1}⊕m_{2}(A)=\frac{1}{1-K}\sum_{\begin{array}{c} B,C\subseteq \Theta ,\\ B\cap C=A \end{array}}m_{1}(B)m_{2}(C),\forall A\subseteq \Theta ,A\neq \phi$$ (5)

$$K=\sum_{B,C\subseteq \Theta ,B\cap C=\phi }m_{1}(B)m_{2}(C)$$

However, if the conflict is high [25], a counter-intuitive result may occur during a combination [12]. As the modification, two main approaches have been introduced: conflict redistributing [26], and discounting unreliable approaches [12], where in our previous study, we had a review on the main discounting approaches [9].

### 2.2 Fuzzy Dempster-Shafer Theory

Since proposal of the concept of fuzzy set by Zadeh [27], the analysis of fuzzy-valued data have rapidly paved a progressive trend due to its importance [28]. To manage imprecise and vague information in evidential reasoning, researchers have tried to generalize the DST in order to deal with fuzzy sets [16] [18] [20] [29]. Fuzzy evidence theory extends DST allowing the assignment of degrees of belief to ambiguous propositions such as those typically expressed in verbal statements which are represented by fuzzy subsets of the FoD.

A fuzzy body of evidence can be defined by the following set:

$$\{<{\tilde{A}}_{j},m({\tilde{A}}_{j}),\mu_{{\tilde{A}}_{j}}>{\}}_{j=1:f}$$ (6)

where Θ is the Frame of Discernment (FoD), *m*(.)is a BPA. Each ${\tilde{A}}_{j}$ is a FE, a normal fuzzy set such that $S({\tilde{A}}_{j})\subseteq \Theta$. $S({\tilde{A}}_{j})$ is the support of a fuzzy set ${\tilde{A}}_{j}$ which it means a crisp set that contains all such points *x*∈Θ for which $\mu_{{\tilde{A}}_{j}}(x)>0$. In the classical set theory, an element *x*∈Θ can either be a member of a certain set *A*⊂Θ or not be a member of this set. The concept of fuzzy set, assumes that *x* can be a member of a fuzzy set $\tilde{A}$ with a certain grade of membership $\mu_{{\tilde{A}}_{j}}(x)$. This grade of membership is defined by the membership function $\mu_{\tilde{A}}:\Theta \to [0,1]$. Therefore, the FBoE framework permits representing all the three types of uncertainty about the quantity the members of Θ. Non-specificity via ${\tilde{A}}_{j}$, fuzziness via $\mu_{{\tilde{A}}_{j}}$, and conflict via $m({\tilde{A}}_{j})$.

Combining multiple fuzzy evidence structures can be extended through the Dempster’s rule if the intersection of crisp sets is replaced by the intersection of fuzzy sets. Following this idea, several rules have been proposed by extending the Dempster’s rule to belief structure through various modalities; Ishizuka et al. considered the intersection degree of two fuzzy sets [16], Yen used two operators with a cross-product operation and normalization process [30], and Yang et al. constructed a weighted variable as follows [31]:

$$m_{1}⊕m_{2}(C{)}_{Yang}=\frac{\sum_{A\cap B=C}W(C,A)m_{1}(A)W(C,B)m_{2}(B)}{1-\sum_{A\cap B\neq \varphi }\left(1-W(A\cap B,A))W(A\cap B,B)m_{1}(A)m_{2}(B\right)}$$ (7)

where, $W(C,A)=\frac{\left|C\right|}{\left|A\right|}$ expresses the weight of contribution to the fuzzy set *C* from a FE *A*.

## 3. Information-based dissimilarity assessment in DST

Dissimilarity assessment is a main problem in DST where quantifying the difference between two BoEs has a central role in many practical algorithms. More than 60 dissimilarity measures have been used for DST in different applications. The idea of multi-dimension dissimilarity measures was first proposed by Liu as a one-dimensional measure is inadequate to quantify the conflict between BoEs [32]. With a focus on different formal properties of dissimilarity measures, Jousselme and Maupin reviewed various dissimilarity measures in DST and selected 15 dissimilarity measures. They classified them into four categories that are metric, pseudo-metric, semi-pseudo-metric and non-metric classes [33]. However, they could not introduce one dissimilarity measure as a sole representor of a class, rather the dissimilarity measures belong to each proposed class were found highly correlated. We developed a methodology that selects a set of dissimilarity measures with the most discrimination power [22]. We investigated almost all studied measures, following a proposed criterion, we used a forward selection procedure to obtain a set of measures that were maximally uncorrelated, while keeping the discrimination as high as possible. The Outcome was the set of most Discriminative Dissimilarity Measures (smDDM) as:

$$SmDDM=\left\{d_{G},\;d_{CS},\;d_{Int},\;d_{E},\;d_{TC},\;d_{N},\;d_{T},\;d_{C}\right\}.$$ (8)

These members are classified into two classes: 1) intrinsic class, enlisting the imperfection of information provided by the sources of evidence ({*d*_*G*_, *d*_*E*_, *d*_*TC*_, *d*_*N*_, *d*_*C*_}), and 2) extrinsic class, enlisting the conflict/contradiction between BoEs ({*d*_*CS*_, *d*_*Int*,_ *d*_*T*_}), where the dissimilarities are measured based on the following criteria [34] [35] [1] [36] [37] [36] [4] [38]:

$$C_{1}=C_{G}(m_{i})=-\sum_{A\subseteq \Theta }m_{i}(A)\log_{2}m_{i}(A).$$ (9)

$$C_{2}=C_{CS}(m_{i})=\frac{1}{\left(M-1\right)}\sum_{i=1,j\neq i}^{M}1-(\frac{{m_{i}}^{\prime }m_{j}}{{\left\|m_{i}\right\|}_{I}.{\left\|m_{j}\right\|}_{I}}).$$ (10)

$$C_{3}=C_{Int}(m_{i})=\frac{1}{\left(M-1\right)}\sum_{i=1,j\neq i}^{M}{m_{i}}^{\prime }(1-Int)m_{j},$$

Int(A,B)=1ifA∩B≠ϕand0otherwise. (11)

$$C_{4}=C_{E}(m_{i})=-\sum_{A\subseteq \Theta }m_{i}(A)\log_{2}Pl(A).$$ (12)

$$C_{5}=C_{TC}(m_{i})=\sum_{A\subseteq \Theta }m_{i}(A)\sum_{B\subseteq \Theta }\left(m_{i}(B)(1-\frac{\left|A\cap B\right|}{\left|A\cup B\right|})\right).$$ (13)

$$C_{6}=C_{N}(m_{i})=1-(\sum_{A\subseteq \Theta ,A\neq \phi }\left(\frac{m_{i}(A)}{\left|A\right|}\right)).$$ (14)

$$C_{7}=C_{T}(m_{i})=\frac{1}{\left(M-1\right)}\sum_{i=1,j\neq i}^{M}\underset{A\subseteq \Theta }{max}\{BetP_{i}(A)-BetP_{j}(A)\},$$

$$BetP(A)=\sum_{B\subseteq \Theta }m(B)(\frac{\left|A\cap B\right|}{\left|B\right|}).$$ (15)

$$C_{8}=C_{C}(m_{i})=-\sum_{A\subseteq \Theta }m_{i}(A)\log_{2}Bel(A).$$ (16)

The results in [22], [5], and [9] showed that the smDDM can be an appropriate and justifiable disagreement measure for various applications. A more efficient description of the difference between two BoEs is obtained when these measures are considered simultaneously. However, they could just handle different types of non-specificity, and conflict (i.e. strife, imprecision, and disparity). In following (Section 4), we extend the smDDM to compare all aspects of information (i.e. fuzziness, non-specificity, and conflict) conveyed by fuzzy bodies of evidence.

## 4. Motivation of the work

The aim of our approach is to find a set of measures of handling different types of uncertainty between two FBoEs, i.e. fuzziness, non-specificity, and conflict. Although the smDDM can appropriately address non-specificity and conflict, the measuring of fuzziness has not been included in it. Moreover, it cannot be used for fuzzy framework. To tackle these challenges, first, we introduce the measures in the fuzzy evidence framework (Section 4.1), then extend the smDDM to apply for the FBoEs (Section 4.2), and add these extended measures to the previously introduced ones to ultimately find a set that could handle different types of uncertainty. However, there exist some redundant information content among these criteria. To find the most important criteria, a backward elimination procedure is proposed which will be discussed subsequently (Section 4.3).

### 4.1. Uncertainty measure in the fuzzy evidence framework

For the fuzzy evidence framework, two main measures have been proposed [39]. The first measure is the General Uncertainty Measure (GM) was introduced by Liu [31].

$$GM(FBoE)\equiv -\sum_{x\in \Theta }\left[BetP(x)\log_{2}BetP(x)+\bar{BetP}(x)\log_{2}\bar{BetP}(x)\right]$$ (17)

where,

$$BetP(x)\equiv \sum_{i=1}^{f}\frac{m({\tilde{A}}_{i})\mu_{{\tilde{A}}_{i}}(x)}{\sum_{x^{\prime }\in S_{{\tilde{A}}_{i}}}\mu_{{\tilde{A}}_{i}}(x^{\prime })},\bar{BetP}(x)\equiv \sum_{i=1}^{f}\frac{m({\tilde{A}}_{i})(1-\mu_{{\tilde{A}}_{i}}(x))}{\sum_{x^{\prime }\in S_{{\tilde{A}}_{i}}}\mu_{{\tilde{A}}_{i}}(x^{\prime })}.$$

The second one is the Hybrid Entropy (FH), proposed by Zhu and Basir [40]. It is defined as a measure which quantifies the overall uncertainty contained in a fuzzy evidence structure:

$$FH(FBoE)\equiv -\sum_{i=1}^{f}m({\tilde{A}}_{i})\log_{2}\left(m({\tilde{A}}_{i})(1-F({\tilde{A}}_{i}))\right)$$ (18)

where $F(\tilde{A})$ denotes the fuzzy entropy of fuzzy set $\tilde{A}$ as:

$$F(\tilde{A})\equiv \frac{1}{\left|S_{\tilde{A}}\right|}\sum_{x\in S_{\tilde{A}}}\frac{\mu_{\tilde{A}\cap \bar{\tilde{A}}}(x)}{\mu_{\tilde{A}\cup \bar{\tilde{A}}}(x)}$$

The smaller is $F(\tilde{A})$, the less fuzzy is the fuzzy set $\tilde{A}$. In Eq (17) and Eq (18), $S({\tilde{A}}_{j})$—the support of a fuzzy set ${\tilde{A}}_{j}$- means a crisp set that contains all such points *x*∈Θ for which $\mu_{{\tilde{A}}_{j}}(x)>0$.

These two measures are considered as the extension of the ambiguity measures (i.e. non-specificity and conflict) to fuzzy set. Therefore, they could only handle types of non-specificity and conflict in fuzzy evidence theory.

Moreover, these measures have been studied through a Monte Carlo simulation [39]. The results have previously revealed while GM behaves with more stability, the FH has counter-intuitive behavior in reflecting the changes in the conflict in FBoEs. Although both GM and FH claimed that quantified aggregate uncertainty is an extension of the ambiguity measure (non-specificity and conflict) to fuzzy set, the differentiation of the quantity of the three types of uncertainty, i.e. fuzziness, non-specificity, and discord is not possible.

There are also some criteria which try to measure fuzziness in an FBoE. A brief summary about fuzziness measures of fuzzy sets has been provided in [41]. The fuzziness of an FBoE is estimated as a weighted sum of fuzziness over all different FEs of FBoE as follows:

$$FM(FBoE)\equiv \sum_{j=1}^{f}m({\tilde{A}}_{j})FE({\tilde{A}}_{j})$$ (19)

where the $FE({\tilde{A}}_{j})$ is a function to measure the fuzziness of each fuzzy set, which can be measured using the quantity proposed by De Luca and Termini [29];

$$FE(\tilde{A})\equiv -\sum_{x\in S_{\tilde{A}}}\mu_{\tilde{A}}(x)\log_{2}\mu_{\tilde{A}}(x)+(1-\mu_{\tilde{A}}(x)\log_{2}(1-\mu_{\tilde{A}}(x))$$ (20)

where $\mu_{\tilde{A}}(x_{i})$ are the fuzzy values, and $S({\tilde{A}}_{j})$—the support of a fuzzy set ${\tilde{A}}_{j}$- means a crisp set that contains all such points *x*∈Θ for which $\mu_{{\tilde{A}}_{j}}(x)>0$. This measure is corresponding to Shannon’s probabilistic entropy whose extended versions can be found in the provided references [42], [43], [44] and [45].

### 4.2. Generalizing the smDDM to fuzzy DST (FsmDDM)

To extend the smDDM for an FBoE, the FBoE should be replaced by BoE. A natural way to link a piece of fuzzy evidence with crisp evidence is to represent the fuzzy set by its α-cuts using the resolution identity principle [35], and then distribute ${\tilde{A}}_{j}$’s mass ($m({\tilde{A}}_{j})$) to its α-cuts. This process can be described as follows:

1. *Decompose the fuzzy FE to its α-cuts*.
  For each α∈[0,1],
  $$A_{j_{\alpha_{k}}}\{x\in X:\mu_{{\tilde{A}}_{j}}(x)\geq \alpha_{k}\}$$ (21)
2. *Distribute the mass of fuzzy FE to its α-cuts*.
  $$m({\tilde{A}}_{j_{\alpha_{k}}})=\frac{m({\tilde{A}}_{j})}{\underset{x}{max}\mu_{{\tilde{A}}_{j}}(x)}(\mu_{{\tilde{A}}_{j}}(x_{k})-\mu_{{\tilde{A}}_{j}}(x_{k+1}))$$ (22)
  which ensures that $m({\tilde{A}}_{j})=\sum_{k}m({\tilde{A}}_{j_{\alpha_{k}}})$

Using smDDM in classical DST, dissimilarity measures can be calculated yielding to have a collection of them for FsmDDM. Taking the average of these measures, a FsmDDM will eventually be obtained which measure all aspects of information between two FBoEs.

### 4.3. Finding the most important criteria

Up to now, we separately estimated the ambiguity and fuzziness with a given FBoE. We add these extended measures to the previously introduced ones (i.e. GM in Eq (17), and FH in Eq (18)) to find a more riche set of measures. Moreover, the addition of the fuzziness measure (i.e. FM in Eq (19)), results a vector which could better handle different types of uncertainty. However, among these criteria, some may have redundant information content.

To find the most important criteria, a backward elimination procedure is proposed to eliminate the lower significant criterion. The advantage of backward elimination is that it gives the opportunity to look at all criteria before removing the least salient one. Finally, we opt a comprehensive set of criteria.

To apply backward elimination, a sample of possible values used on all criteria should be selected. In the first step, 1000 random FBoEs is generated by adapted algorithm which is proposed in [39] based on Tessem’s idea [4]. ‎Algorithm I summarize the generating procedure.

## Algorithm I. Generatinging a random FBoE

Definition

    f: Number of Focal Elements (FEs)

    Θ: Frame of Discernment (FoD)

while *f* > 0,

    *rest* = 1

    for *i* = 1 to *f-1* do

        Generate a random number *X*

        Generate a normal convex fuzzy set ${\tilde{A}}_{i}$ with $\mu_{{\tilde{A}}_{i}}$ on Θ

    $m({\tilde{A}}_{i})=P(Y\leq X).rest$

    *rest* = *rest* -$m({\tilde{A}}_{i})$

    end

    Generate a normal convex fuzzy set ${\tilde{A}}_{f}$ with $\mu_{{\tilde{A}}_{f}}$ on Θ

    $m({\tilde{A}}_{f})=rest$

end while

Then, we assess each FBoE based on all criteria (i.e. FsmDDM, GM, FH, and FM). As a result, a source value is obtained. The backward elimination starts with 4 criteria and removes the criterion with least information in each step as follows:

$$j^{*}=\arg\;\min_{\begin{array}{c} j\in remained\;criteria\\ j\neq k \end{array}}\min_{k\in \mathrm{all}\;\mathrm{critera}}\left[\frac{1}{{\left\|{Criterion}_{j}\right\|}_{1}}{\left\|{Criterion}_{j}-\frac{<{Criterion}_{j},{Criterion}_{k}>}{<{Criterion}_{k},{Criterion}_{k}>}{Criterion}_{k}\right\|}_{1}\right]$$ (23)

In each step of backward elimination, the normalized distance between the *j*th criterion and its orthogonal projection on the *k*th criterion, since the *j*th criterion has more independent information content related to other criteria, the scoring criterion increases. In the worst case, the minimum of this scoring criterion for all criteria is taken in J(*j*).

**Fig 1:**
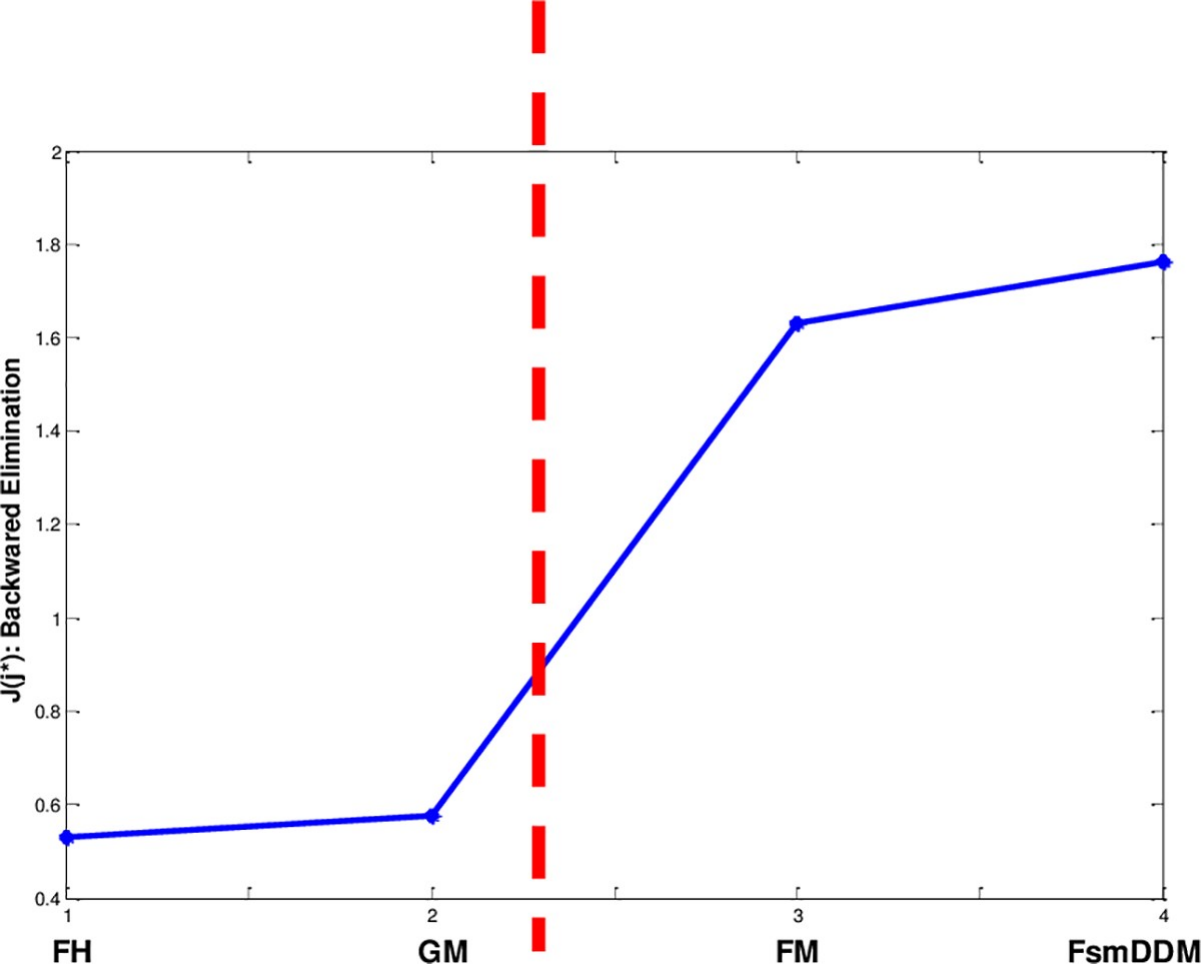
Score of selection. Removing the two criteria yields not much unimportant criteria.

Fig 1 shows the score of selection in order of removal, where 4 criteria (i.e. FsmDDM, GM, FH, and FM) are considered. After removing FH, and GM, the graph increases. This means that choosing the first two removed criteria does not convey much more information. As a result, the FsmDDM along with the fuzzy measure (i.e. FM) result a vector which could better handle different types of uncertainty.

## 5. Result and discussion

Due to lack of available experimental data from real application in the literature for the fuzzy DST framework, we evaluate the FsmDDM as well as FH, and GH through a simulation-based analysis. The obtained results for the measures are compared and their accuracy and reliability are being discussed in the following.

### 5.1. Difference between measures through simple examples

In order to examine the behavior of the dissimilarity measures, we first generate simple numerical examples of FBoEs, which allow us to predict changing of the content of information and to compare the corresponding values obtained by different measures.

Assuming three fuzzy bodies of evidence as follows:

$${FBOE}_{1}\equiv \left\{1/0.49,2/1,3/0.49\right\}/0.5,\;\left\{1/0.49,2/1,3/0.49\right\}/0.5.$$

$${FBOE}_{2}\equiv \left\{1/0.5,2/1,3/0.5\right\}/0.5,\;\left\{1/0.5,2/1,3/0.5\right\}/0.5.$$

$${FBOE}_{3}\equiv \left\{1/0.5,2/1,3/1\right\}/0.5,\;\left\{4/1,5/1,6/1\right\}/0.5.$$

It can be seen that the fuzziness is slightly smaller in *FBOE*_1_ as compared to the other two, but remains the same for the two others. The discord, however, is almost the same for *FBOE*_1_ and *FBOE*_2_ while it dramatically changes from *FBOE*_2_ to *FBOE*_3_. Here are the values of measures for these BoEs.

$${GM(FBOE}_{1})=2.50\;{GM(FBOE}_{2})=2.50\;{GM(FBOE}_{3})=4$$

$${FH(FBOE}_{1})=2.48\;{FH(FBOE}_{2})=2.58\;{FH(FBOE}_{3})=2.58$$

$${FM(FBOE}_{1})=1.99\;{FM(FBOE}_{2})=2\;{FM(FBOE}_{3})=2$$

$${FsmDDM(FBOE}_{1})=0.203\;{FsmDDM(FBOE}_{2})=0.204\;{FsmDDM(FBOE}_{3})=0.48$$

As observed, the results show that the FsmDDM could be a better measure in reflecting changes in all types of information.

### 5.2. Difference between measures through average behavior during combination of EBoEs

**Fig 2:**
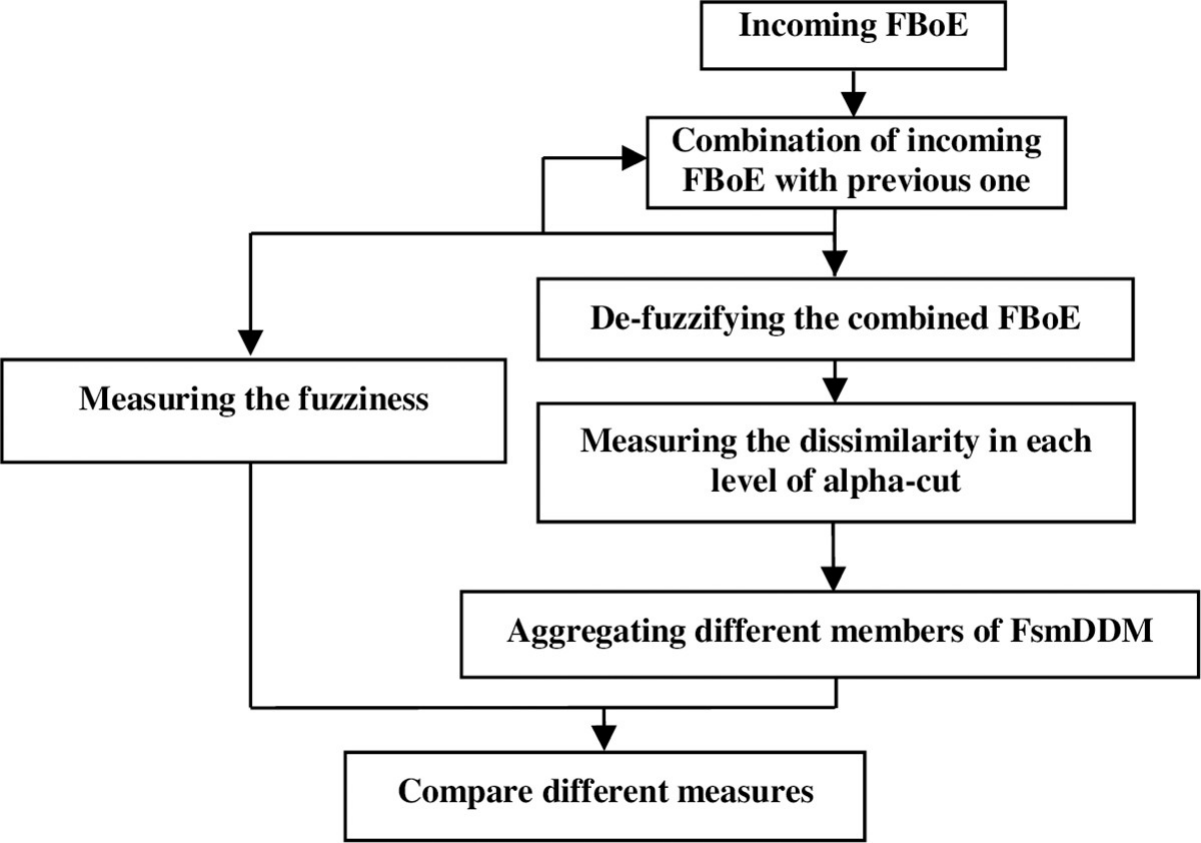
Information-based comparison approach during combination process between two fuzzy bodies of evidence.

We use a Monte Carlo simulation, as previously used in similar works [39], in a combination procedure. The process of combining can reflect the uncertainty decrease when the multiple FBoEs are combined sequentially. Fig 2 shows the procedure of combination process and information-based comparison in general.

**Fig 3:**
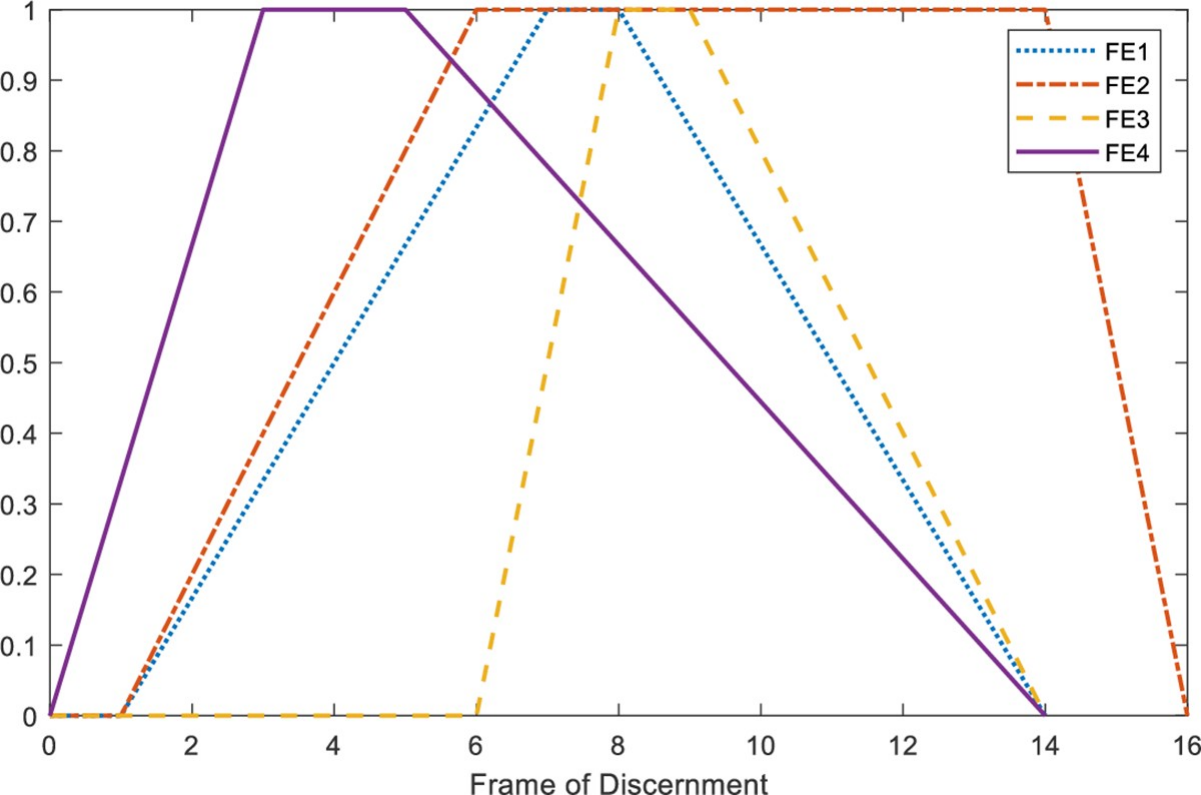
A FBoE with four normal trapezoidal fuzzy focal elements.

A random FBoE is generated by adapted algorithm which is proposed in ‎Algorithm I. In our experiment, |*Θ*| = 16, four fuzzy FEs for each new incoming FBoE are considered. Fig 3 illustrates one FBoE with four normal trapezoidal fuzzy numbers as its FEs, which is randomly generated through Algorithm I.

During the experiment, 20 combinations are assumed. In order to combine the information provided by each piece of evidence in fuzzy DST, we use the Yang et al. combination’ rule as Eq (7). To compute the FsmDDM, the new combined FBoE has to be normalized fuzzy set. Here, we used the normalization method proposed by Yager and Filev [46]. Their method is based on the scaling up. The normalization process is presented in Algorithm II.

## Algorithm II. Normalization process

    Definition

    FBoE: $\{<{\tilde{A}}_{j},m({\tilde{A}}_{j}),\mu_{{\tilde{A}}_{j}}>{\}}_{j=1:f}$, *T* = 1;

    for *i* = 1 to *f* do

        $v_{i}=\underset{x}{max}\mu_{{\tilde{A}}_{i}}(x)$

        $u_{i}=m({\tilde{A}}_{i})v_{i}$

        *T* = *T*−*u_i_*

        $\forall x\in \Theta ,\mu_{{\tilde{A}}_{i}}(x)=\frac{\mu_{{\tilde{A}}_{i}}(x)}{v_{i}}$

    end

    for *i* = 1 to *f* do

        $m({\tilde{A}}_{i})=\frac{u_{i}}{1-T}$

    end

**Fig 4:**
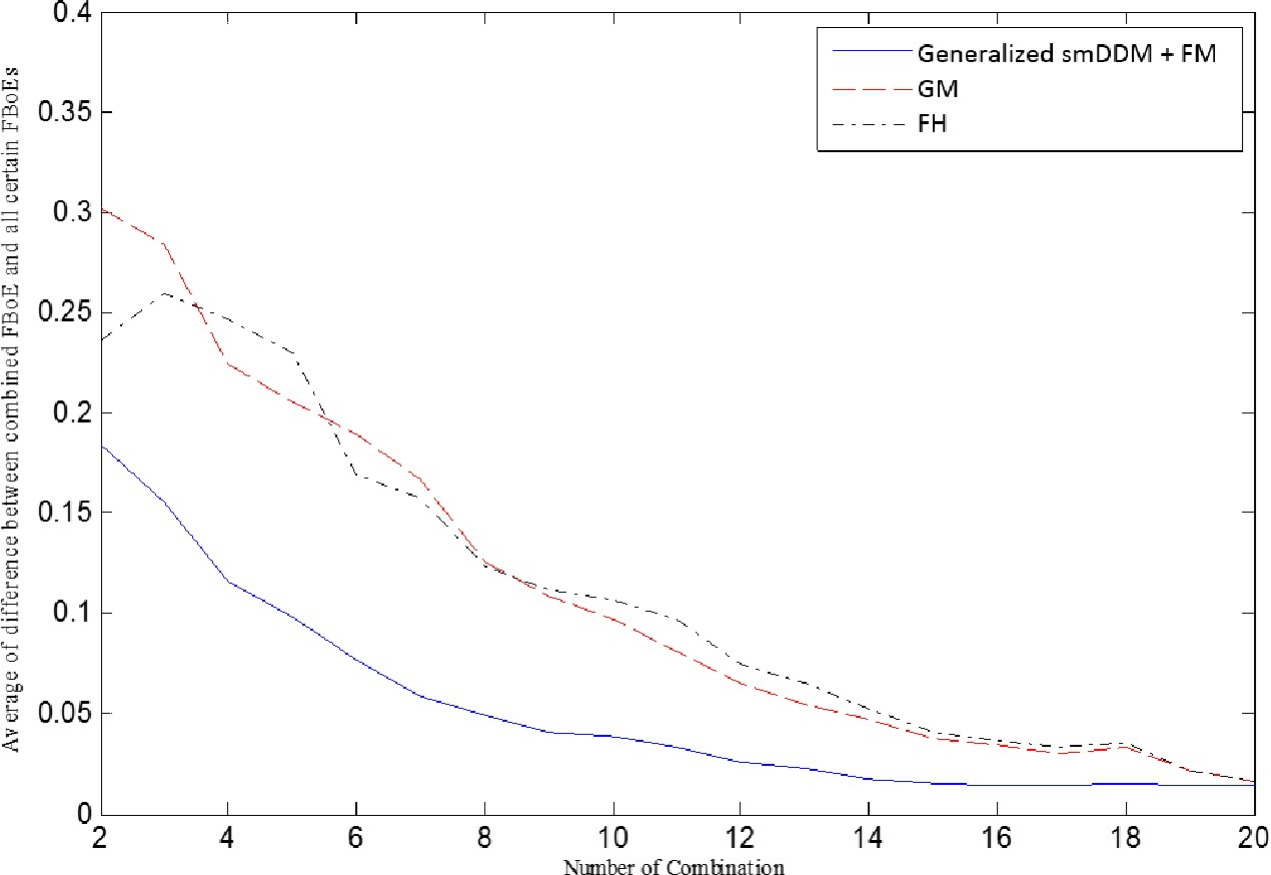
Mean of dissimilarity measures, when the combined FBoE is compared with all certain FBoEs, here the dash line shows the mean of FsmDDM plus fuzziness measure.

In each combination step, the difference between combined FBoE with the previous FBoE is measured as follows:

$$d_{U}(m_{1},m_{2})=|U(m_{1})-U(m_{2})|$$ (24)

where, *U* can be any measure. Then, the experiment is repeated 50 times and the mean of differences is calculated. Fig 4 shows the resulting curves. The results are in line with the results reported in [39], here, although GM is more stable that FH, the behavior between two consecutive combination steps does not decrease all the time. As shown in Fig 4, our proposed overall dissimilarity based on FsmDDM has more stable behavior between two consecutive combination steps in the sense that the average curve of our proposed dissimilarity is more stable than GM and FH. However, the aggregate uncertainty measures of GM and FH have no acceptable behavior during combination.

## 6. Conclusion

Fuzzy evidence theory captures all three types of uncertainty; fuzziness, non-specificity, and conflict, that are usually contained in a piece of information within one framework. Quantifying the difference between two FBoEs plays a central important role when this framework is used in applications.

In this paper, we have proposed a method to compare two FBoEs comprehensively. Although our previously proposed smDDM could be an appropriate approach to handle non-specificity and conflict, the measuring of fuzziness was not included in it. Moreover, the smDDM could not be used for fuzzy framework. In our approach, the smDDM has been extended to apply for FBoEs, then it has been added to the previously introduced ones, in order to find a rich set of measures capable of handling different types of uncertainty. To eliminate the redundant information, a backward elimination process has been employed to find the most important criteria. In the proposed backward selection procedure, a scoring criterion has been designed to gradually remove unimportant DMs. Eventually, a set of most important ones has been selected and its effectively has been discussed in details. Consequently, we have obtained a set of dissimilarity measure which can measure all three types of uncertainty: fuzziness, non-specificity, and conflict.

The ability and stability of the proposed measure through a Monte-Carlo simulation have been investigated extensively. The results show that the trend curves have an excellent acceptable behavior when the FsmDDM is used.
